# Green enrichment of α-terpineol from niaouli oil residue *via* molecular distillation

**DOI:** 10.1039/d6ra00953k

**Published:** 2026-05-14

**Authors:** Dinh-Nhat Do, Minh-Tinh Trinh-Le, Hoang-Yen Nguyen-Thi, Minh-Chau Pham-Vu, Tan-Viet Tran, Xuan-Tien Le

**Affiliations:** a Faculty of Chemical Engineering, Ho Chi Minh City University of Technology (HCMUT) 268 Ly Thuong Kiet Street, Dien Hong Ward Ho Chi Minh City Vietnam tien.le@hcmut.edu.vn; b Vietnam National University Ho Chi Minh, (VNU-HCM) Linh Xuan Ward Ho Chi Minh City Vietnam; c Faculty of Applied Science and Technology, Nguyen Tat Thanh University Ho Chi Minh City Vietnam

## Abstract

The bottom fraction, obtained as a residual by-product after commercial 1,8-cineole recovery from the fractional distillation of *Melaleuca quinquenervia* essential oil, is often underutilized despite its potential as a source of valuable bioactive compounds. In this study, the residue was recovered and further purified using molecular distillation to enhance the concentration of α-terpineol. Key processing parameters, including pressure (Pa), evaporator surface temperature (°C), and feed flow rate (mL min^−1^), were systematically investigated and optimized using response surface methodology (RSM) to maximize α-terpineol content. The optimal conditions were determined to be 209.7 Pa, 35.4 °C, and 1.39 mL min^−1^, resulting in an α-terpineol content of 70.97% with a recovery rate of 89.2%. GC–MS analysis confirmed that molecular distillation effectively concentrated α-terpineol in the light fraction, while valuable oxygenated sesquiterpenes were enriched in the heavy fraction. Antibacterial activity, evaluated by the agar disk diffusion method, demonstrated that the α-terpineol-enriched light fraction was active against both Gram-positive and Gram-negative bacteria, with efficacy comparable to ampicillin. Anti-inflammatory activity was further confirmed by dose-dependent inhibition of nitric oxide production in LPS-stimulated RAW 264.7 macrophages, while high cell viability indicated low cytotoxicity. These findings demonstrate that molecular distillation provides an effective and solvent-free approach for sustainably valorizing the bottom fraction of *M. quinquenervia* essential oil into bioactive fractions.

## Abstract 2

Molecular distillation enabled green enrichment of α-terpineol from niaouli oil residue, transforming an underutilized by-product into a bioactive fraction with enhanced antibacterial and anti-inflammatory potential.

## Graphical abstract

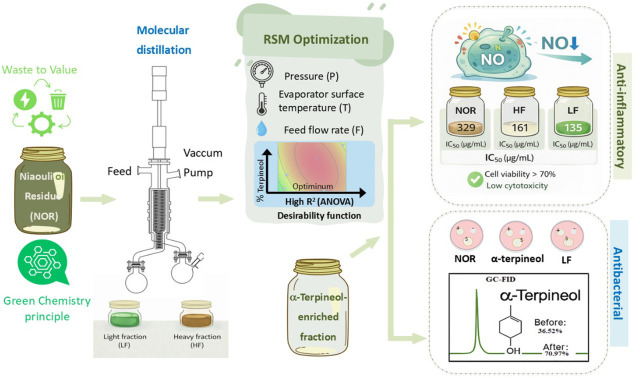

## Introduction

*Melaleuca quinquenervia* (Cav.) S.T. Blake, commonly known as “niaouli”, belongs to the Myrtaceae family, a large plant family predominantly native to Australia.^1,2^ In Vietnam, this species is widely distributed across diverse ecological regions and serves as an important raw material for the essential oil industry. The essential oil of *M. quinquenervia* is primarily obtained from the leaves by steam distillation.^1^ Its chemical composition varies with geographical origin and chemotype, with two major chemotypes identified: a nerolidol-rich type and a 1,8-cineole-dominant type.^3^ In Vietnam, the oil typically contains a high proportion of 1,8-cineole, together with other bioactive constituents such as α-terpineol, viridiflorol, limonene, α-pinene, and β-caryophyllene.^4^ Owing to this composition, niaouli essential oil exhibits a range of biological activities, including antibacterial, anti-inflammatory, antioxidant, and insect-repellent effects, highlighting its potential as a natural ingredient for pharmaceutical, cosmetic, and personal care applications.^5^

In *Melaleuca quinquenervia* essential oil, 1,8-cineole is the major component and is considered the primary contributor to the oil's biological activities.^6,7^ Currently, vacuum fractional distillation is commonly employed to isolate 1,8-cineole for commercial applications. After separation and purification, the heavy residue remaining at the bottom of the distillation column is often discarded or left unutilized. However, this residue still contains several valuable compounds, most notably α-terpineol, which plays an important role in the fragrance, cosmetic, and food industries.^8^ In addition, α-terpineol has gained significant interest due to its diverse biological activities, including antioxidant,^9^ anti-inflammatory,^10^ anticonvulsant,^11^ anticancer,^12^ and particularly antimicrobial properties.^13^ Given that this residue is rich in high-boiling compounds, novel techniques are required to separate and purify these constituents as potential raw materials for the pharmaceutical and cosmetic industries, thereby enhancing the overall value of the essential oil.

In recent years, molecular distillation (MD) has emerged as a specialized technique for separating heat-sensitive or high-boiling-point liquid mixtures.^14^ The MD apparatus is designed with a very short distance—typically only a few centimeters—between the evaporator and the condenser, and it operates under high-vacuum conditions. During operation, the feed material is distributed as a thin film over the heated evaporator surface, ensuring uniform heat transfer and efficient evaporation. Owing to the low operating pressure and residence times of only a few seconds, volatile components can be separated at significantly lower temperatures than those required in conventional distillation, thereby minimizing thermal degradation.^15,16^ The separation mechanism of molecular distillation is not governed solely by differences in boiling points but is also closely related to the concept of mean free path, defined as the average distance a molecule travels before a collision alters its direction or energy.^17,18^ Accordingly, the distance between the evaporator and the condenser is designed to be comparable to or smaller than the mean free path at the operating pressure, allowing volatile molecules to travel directly to the condensation surface with minimal intermolecular collisions, thereby enhancing separation efficiency.^18^

**Fig. 1:**
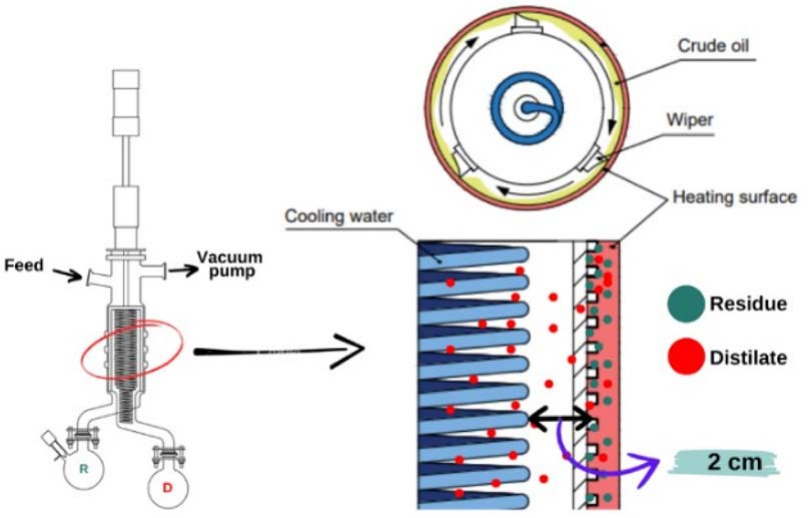
Operating principle of molecular distillation.

In this study, the niaouli oil residue (NOR) is the bottom fraction remaining after vacuum fractional distillation of niaouli essential oil, in which higher-boiling oxygenated terpenoids are enriched. Further recovery of these compounds by conventional distillation (*e.g.*, fractional distillation, even under reduced pressure) becomes inefficient due to the high temperatures and prolonged heating times required, as well as the associated risk of thermal degradation. Therefore, molecular distillation was employed as a suitable technique to recover and enrich oxygenated terpenoids from the residue, with α-terpineol as the target compound. During molecular distillation, the feed was spread as a thin film over the heated evaporator surface. The light fraction (LF, distillate) condensed on the cooled surface located a short distance from the evaporator and was collected in the distillate receiver, whereas the heavy fraction (HF, residue) was retained in the residue flask^17,19^ (Fig. 1).

Consequently, MD is regarded as a non-equilibrium separation process, characterized by short heating times and high vacuum levels, which enable the recovery of target compounds while minimizing thermal degradation of sensitive constituents.^20,21^ This feature makes MD particularly advantageous for preserving essential oil quality. Several studies have demonstrated the successful application of MD in purifying essential oils such as lemongrass,^22^ basil,^23^ oregano,^24^ rose,^25^ grapefruit,^26^ citrus,^27^ and rosemary.^28^ MD is a modern separation method capable of purifying and fractionating essential oils into high-purity target compounds. It meets the quality demands of the pharmaceutical and food industries, which require strict control over raw materials and reduce potential toxicity associated with solvent-based extraction methods. However, it remains a complex process involving multiple variables.^29,30^ Although introduced decades ago, its application in practice remains limited, and further research is needed to support its broader implementation.

Therefore, this study aimed to recover and enrich α-terpineol from the NOR obtained after vacuum fractional distillation of *Melaleuca quinquenervia* essential oil by applying MD. The effects of three key operating parameters—pressure, evaporator surface temperature, and feed flow rate—were systematically investigated and optimized using response surface methodology (RSM) to maximize α-terpineol content and recovery yield. In addition, the biological potential of the TEF was evaluated through antibacterial assays against both Gram-positive and Gram-negative bacteria. Its anti-inflammatory activity was also assessed based on the inhibition of nitric oxide production in LPS-stimulated RAW 264.7 macrophages, thereby supporting its potential for pharmaceutical and cosmetic applications.

## Experimental

### Material

**Fig. 2:**
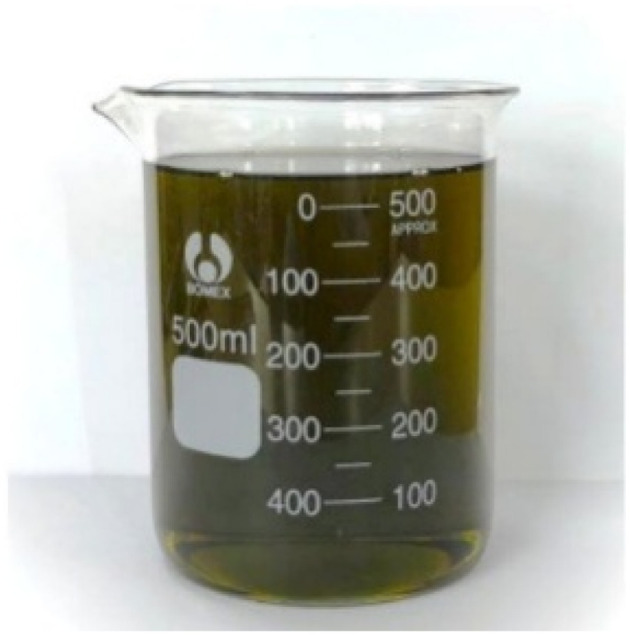
Niaouli oil residue (NOR).

The niaouli essential oil was supplied by Notessen Company Limited, with plant material harvested in Quang Tri, Vietnam. The oil was first subjected to vacuum fractional distillation to isolate 1,8-cineole as the target fraction. The resulting niaouli oil residue (NOR), collected from the bottom of the distillation column (Fig. 2), was subsequently analyzed and further processed by MD to recover α-terpineol in the LF.

### Molecular distillation system

#### Apparatus

**Fig. 3:**
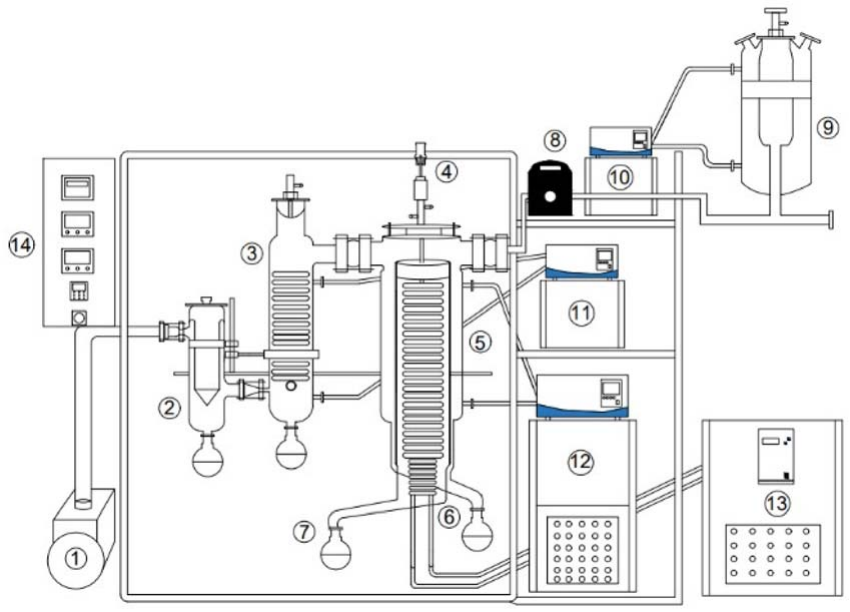
Schematic diagram of the molecular distillation (MD) system.

Fractionation of NOR was carried out using a short-path molecular distillation (MD) system (model AYAN-F60, Hangzhou Anyan Instrument Manufacturing Co., Ltd, Hangzhou, China), equipped with a vacuum pump, peristaltic feed pump, and temperature control units. The evaporator surface area was 0.10 m^2^, the condenser surface area was 0.06 m^2^, and the evaporator-condenser distance was 0.02 m (Fig. 3). The evaporator surface temperature was controlled using a dedicated heating circulator (12), while the condenser temperature was maintained by a refrigerated cooling circulator (11, 13). The feed (9) was delivered at a controlled flow rate using a peristaltic pump (8). The operating pressure was continuously monitored and regulated by a vacuum pump (1) connected to a pressure control unit (14).

#### Operating conditions and experimental variables

Experiments were conducted at a rotor (wiper) speed of 250 rpm and a condenser temperature of 2 °C. Three operating variables were investigated: absolute pressure (Pa), evaporator surface temperature (°C), and feed flow rate (mL min^−1^). These variables were selected due to their direct influence on evaporation flux, residence time, and the thermal stability of the constituents.

### Determination of the chemical composition

#### Qualitative analysis by GC–MS

The NOR, LF and HF were analyzed for qualitative composition by gas chromatography–mass spectrometry (GC–MS) using a TRACE 1310 GC coupled with a TSQ 9000 MS (Thermo Fisher Scientific, USA). Separation was achieved on an HP-5MS capillary column (30 m × 0.25 mm i.d., 0.25 µm film thickness; Agilent). Helium was used as the carrier gas at a flow rate of 1.2 mL min^−1^, with a split ratio of 1 : 100. The injector and ion source temperatures were set at 250 °C and 230 °C, respectively. The oven temperature was programmed from 60 to 240 °C at 3.0 °C min^−1^, then increased to 270 °C at 5.0 °C min^−1^ and held for 2 min. Mass spectra were acquired in electron ionization (EI) mode at 70 eV over a mass range of 50–550 amu with a scan rate of 1 scan per s. Compounds were identified by comparison of their mass spectra with those in the NIST 2.2 and Adams libraries. In particular, α-terpineol was identified with a high match factor (MF = 905), indicating excellent spectral agreement. The relative composition of the detected compounds was calculated by GC peak area normalization and expressed as percentage peak areas.

#### Quantitative analysis by GC-FID

α-Terpineol was quantified using a gas chromatography-flame ionization detection (GC-FID) system (GC-2030, Shimadzu, Japan) equipped with an SH-I-5MS capillary column (30 m × 0.25 mm i.d., 0.25 µm film thickness). Samples were diluted 100-fold with hexane and injected in split mode (1 : 100). Nitrogen was used as the carrier gas at a flow rate of 1.2 mL min^−1^. The injector temperature was set at 250 °C. The oven temperature was programmed from 60 to 240 °C at 3.0 °C min^−1^, then increased to 270 °C at 5.0 °C min^−1^ and held for 2 min. Quantification was performed using a calibration curve of α-terpineol, which showed good linearity with the regression equation *Y* = 156.7*X* − 17934.9 (where *Y* represents GC peak area and *X* represents the concentration of α-terpineol) and a coefficient of determination (*R*^2^ = 0.9996).

#### Recovery of α-terpineol in the light fraction (LF)

The recovery of α-terpineol in the LF was determined on eqn (1):1
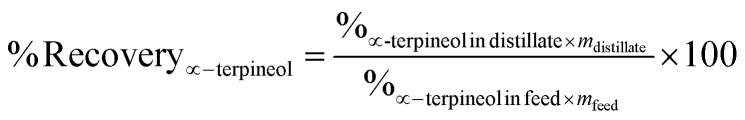


### Optimization of operating parameters using the response surface methodology (RSM)

The response surface methodology (RSM) was applied using Design-Expert software version 13.0 (Stat-Ease Inc., Minnesota, USA) to optimize key operating parameters of the molecular distillation process with the aim of maximizing α-terpineol content. Experimental trials were designed according to a Central Composite Design (CCD) with three independent variables.

The experimental data were fitted to a second-order polynomial model, with the response variable *Y* (α-terpineol content, %) expressed as a function of the independent variables according to the following eqn (2):

2 *Y* = *α*_0_ + *α*_1_*X*_1_ + *α*_2_*X*_2_ + *α*_3_*X*_3_ + *α*_12_*X*_1_*X*_2_ + *α*_13_*X*_1_*X*_3_ + *α*_23_*X*_2_*X*_3_ + *α*_11_*X*_1_^2^ + *α*_22_*X*_2_^2^ + *α*_33_*X*_3_^2^

where *α*_0_ is the intercept term, *α*_1_, *α*_2_, *α*_3_, *α*_12_, *α*_13_, *α*_23_, *α*_11_, *α*_22_, *α*_33_ represent the linear, interaction, and quadratic coefficients estimated by Design-Expert software.

(1) Vacuum pump; (2) vacuum trap; (3) condenser; (4) motor; (5) main distillation chamber (condensing and evaporating surfaces); (6) residue collector; (7) distillate collector; (8) peristaltic pump; (9) feed tank; (10) feed heating circulator (preheating unit); (11) cooling circulator for condenser; (12) evaporator surface temperature control system; (13) cooling circulator for condenser; (14) control cabinet.

### Evaluation of antibacterial activity

Antibacterial activity was evaluated using the agar disk diffusion method (Kirby–Bauer test).^31^ Each agar plate was prepared with 20 mL of Mueller–Hinton Agar (MHA) medium. Five bacterial strains, including *Staphylococcus aureus* (Gram-positive), *Escherichia coli*, *Vibrio parahaemolyticus*, *Klebsiella pneumoniae*, and *Pseudomonas aeruginosa* (Gram-negative), were used in this study. Bacterial suspensions were adjusted to a concentration of 10^6^ CFU mL^−1^, and 25 µL of each suspension was evenly spread over the agar surface. Sterile paper discs (6 mm in diameter, 0.5 mm thick) were placed on the inoculated plates, and 25 µL of each test solution was applied to the discs. Distilled water was used as a negative control, while ampicillin (10 µg per disc) served as the positive control. The plates were incubated at 37 °C for 24 h. Antibacterial activity was evaluated by measuring the diameter of the inhibition zones surrounding each disc, excluding the disc diameter. All experiments were performed in triplicate.

### Evaluation of anti-inflammatory activity

The anti-inflammatory activity was assessed using lipopolysaccharide (LPS)-stimulated RAW 264.7 macrophages (RRID: CVCL_0493, ATCC, USA). Cells were cultured in Dulbecco's modified Eagle medium supplemented with 10% fetal bovine serum and 1% penicillin/streptomycin (Gibco, Carlsbad, CA, USA) and maintained at 37 °C in a 5% CO_2_ atmosphere. Cells were then seeded in 96-well plates at a density of 2 × 10^4^ cells per well. After 24 h, cells were treated with test samples or dexamethasone (positive control) at various concentrations for 1 h, followed by stimulation with LPS (1 µg mL^−1^) for 24 h.

Nitric oxide (NO) production was determined using the Griess reagent method as previously described by Loizzo *et al.*^32^ Briefly, 50 µL of culture supernatant was mixed with 50 µL of Griess reagent in the dark, shaken for 10 min, and the absorbance measured at 540 nm. The inhibition of NO production was calculated on eqn (3):3
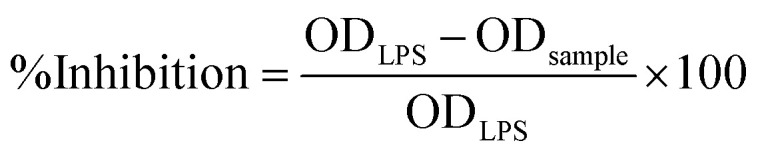


Cell viability was simultaneously evaluated using the 3-(4,5-dimethylthiazol-2-yl)-2,5-diphenyltetrazolium bromide (MTT) assay. After NO measurement, 5 µL of MTT solution (5 mg mL^−1^) was added to each well. Following 3 h incubation at 37 °C in a humidified atmosphere with 5% CO_2_, the medium was removed, and the formazan crystals were dissolved in 100 µL of DMSO. Absorbance was measured at 570 nm, and cell viability was indicated on eqn (4):4
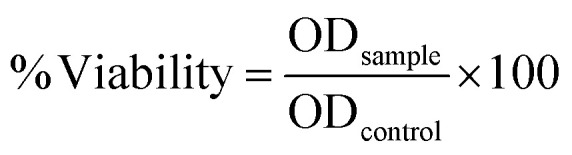


### Statistical analysis of data

Experimental and analytical data were statistically analyzed using Microsoft® Excel® 2021 MSO and IBM SPSS Statistics version 27.0.1. One-way analysis of variance (one-way ANOVA) was performed to evaluate differences among samples and treatments. When significant differences were observed, Tukey's multiple comparison test was applied to determine differences between mean values. Statistical significance was defined at a confidence level of 95% (*p* < 0.05).

## Results and discussion

### Preliminary investigation of influencing factors affecting the light fraction (LF)

In this study, molecular distillation was employed to recover and enrich α-terpineol from the niaouli oil residue (NOR) obtained after vacuum fractional distillation of Melaleuca quinquenervia essential oil. To ensure that subsequent optimization was conducted within appropriate ranges, the effects of three key operating parameters—pressure, evaporator surface temperature, and feed flow rate—were initially investigated. This preliminary assessment provided a basis for defining the operating window of each factor prior to response surface methodology (RSM) optimization.

#### Effect of operating pressure on α-terpineol content and recovery

**Fig. 4:**
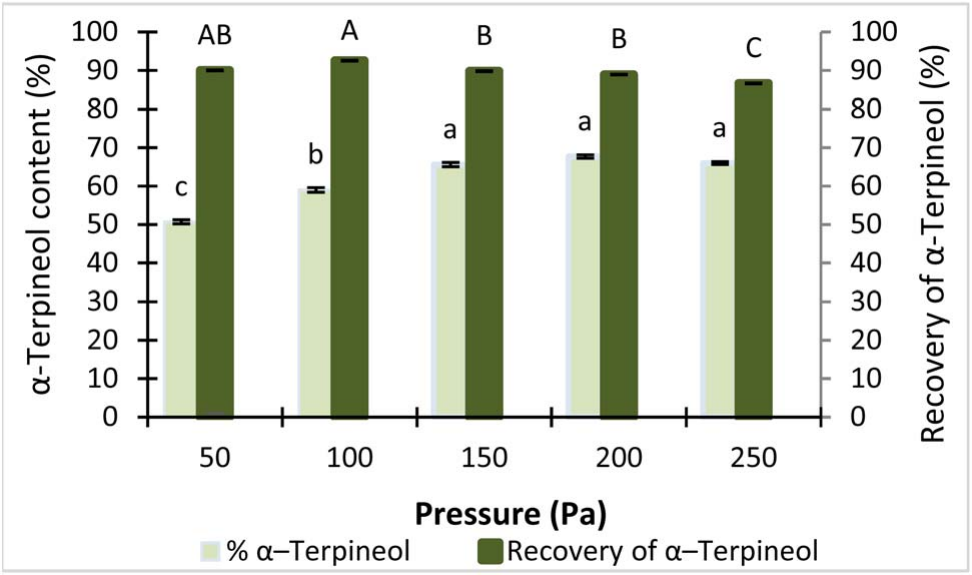
Effect of pressure on the content and recovery of α-terpineol. Bars with different letters are significantly different at *p* < 0.05.

Operating pressure is a critical factor in MD, as it governs the mean free path of vapor molecules and, consequently, the efficiency of mass transfer. At higher vacuum levels (*i.e.*, lower operating pressures), the mean free path of vapor molecules increases. When the mean free path becomes comparable to or exceeds the distance between the evaporator and condenser surfaces, evaporated molecules can travel directly to the condenser surface and be efficiently collected in the LF.^18^ Therefore, the effect of operating pressure in the range of 50–250 Pa on the enrichment of α-terpineol in the LF was investigated (Fig. 4), while other parameters were maintained constant, including an evaporator surface temperature of 35 °C, a feed flow rate of 1.5 mL min^−1^, a rotor speed of 250 rpm, and a condenser temperature of 2 °C.

The NOR used as the feed initially contained 32.5% α-terpineol, as determined by GC-FID analysis. After molecular distillation, the α-terpineol content in the LF increased markedly, as shown in Fig. 4, with a steady increase observed as the operating pressure rose from 50 to 200 Pa, followed by a slight decrease at 250 Pa. Under high-vacuum conditions (50–100 Pa), the mean free path of vapor molecules is sufficiently long to allow not only α-terpineol but also heavier components to reach the condenser, resulting in a lower α-terpineol concentration in the LF. As the pressure increased to the range of 100–200 Pa, heavier components with molecular weights higher than that of α-terpineol were no longer efficiently transported to the condenser, leading to an enrichment of α-terpineol in the light fraction.

This behavior can also be explained by differences in volatility among the components. Under reduced pressure, compounds with higher vapor pressures evaporate more readily and preferentially reach the condenser surface. As the operating pressure increases, the effective separation becomes more selective, limiting the co-evaporation of less volatile, higher-molecular-weight compounds and thereby enhancing the enrichment of α-terpineol in the light fraction. A similar trend was reported by F. Chen *et al.* (2006) during the separation of octacosanol from rice bran wax.^33^ Based on these observations, a pressure range of 150–250 Pa was considered appropriate for subsequent optimization using response surface methodology.

Meanwhile, the recovery of α-terpineol remained relatively high (86.6–92.5%) and nearly constant across the investigated pressure range, with only a slight decrease observed at higher pressures (200–250 Pa). This indicates that operating pressure has a limited influence on the overall recovery of α-terpineol, while having a more pronounced effect on its enrichment in the light fraction. A similar observation has been reported in the molecular distillation of tocopherols from rapeseed oil deodoriser distillate, where operating pressure was shown to significantly influence the fractionation behavior, governing the distribution of components between the distillate and heavier fractions.^34^ To select appropriate operating conditions for subsequent experiments, a pressure of 200 Pa—at which the α-terpineol content reached its maximum value (65.73%)—was fixed as the operating pressure for the system.

#### Effect of evaporator surface temperature on α-terpineol content and recovery

Evaporator surface temperature is a critical parameter in MD, as it directly influences the vapor pressure of compounds and, consequently, their tendency to evaporate.^20^ At low temperatures, insufficient evaporation can lead to incomplete separation and reduced recovery in the light fraction. Conversely, excessively high temperatures may promote the co-evaporation of heavier compounds and increase the risk of thermal degradation of heat-sensitive molecules, thereby decreasing product purity.^35^ In this study, the effect of evaporator surface temperature on the content and recovery of α-terpineol in the light fraction was investigated over a temperature range of 30–50 °C.

**Fig. 5:**
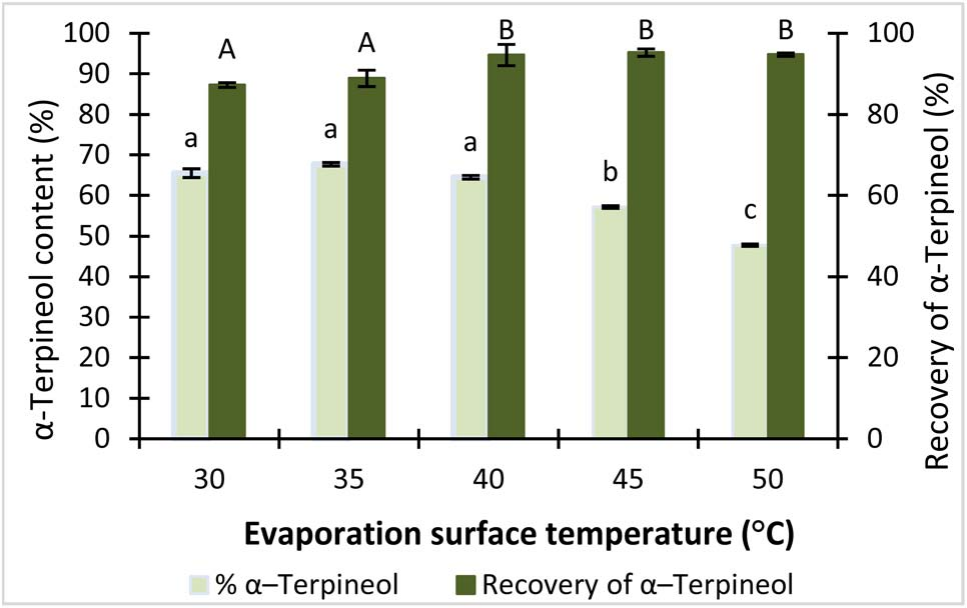
Effect of evaporator surface temperature on the content and recovery of α-terpineol. Bars with different letters are significantly different at *p* < 0.05.

At evaporator surface temperatures of 30 °C and 35 °C, the LF contained relatively high amounts of α-terpineol (65.5% and 67.7%), while recovery yields remained satisfactory at 87.3% and 88.9%, respectively (Fig. 5). Increasing the temperature to 40 °C enabled most α-terpineol molecules to evaporate and condense, resulting in a maximum recovery yield of 94.6%; however, the co-evaporation of other compounds reduced the α-terpineol content to 64.5%. This trend became more pronounced at 45 °C and 50 °C, where recovery yields stayed above 94% but the α-terpineol content in the LF further declined to 57.1% and 47.8%, respectively. These results demonstrate that evaporator surface temperature strongly affects the balance between recovery and purity, consistent with the findings of Tovar *et al.* on the MD of citral-enriched fractions from lemongrass essential oil.^22^ Therefore, an evaporator temperature range of 30–40 °C was selected for subsequent optimization using response surface methodology.

#### Effect of feed flow rate on α-terpineol content and recovery

While pressure and temperature primarily determine the volatility and thermal behavior of compounds, the feed flow rate mainly influences the hydrodynamic conditions of the molecular distillation process. By regulating the thickness and renewal of the liquid film on the evaporator surface, this parameter affects the extent of evaporation and, consequently, the fractionation efficiency.^17^ To examine this balance, feed flow rates of 1.0, 1.5, 2.0, 2.5, and 3.0 mL min^−1^ were systematically investigated to evaluate their effects on the α-terpineol content and recovery in the LF.

**Fig. 6:**
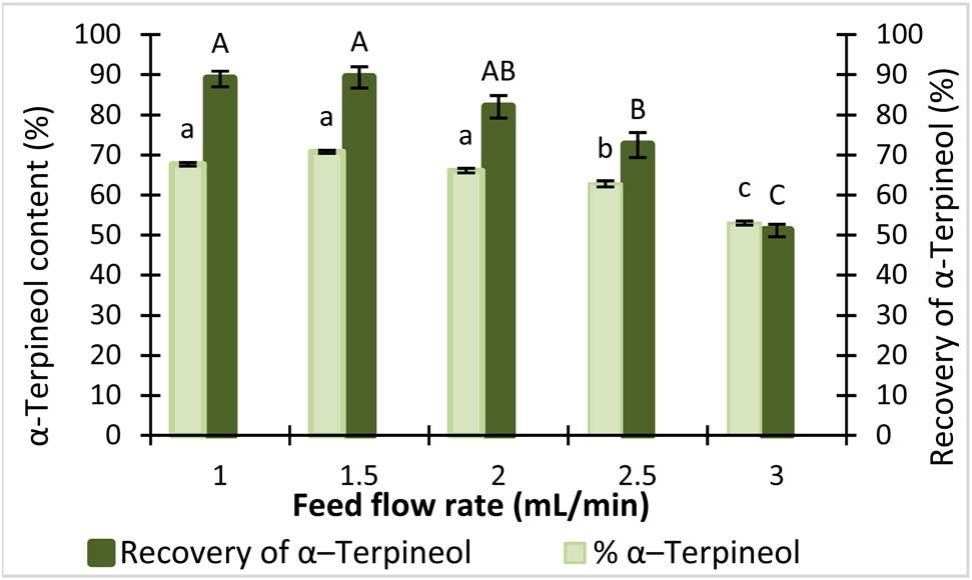
Effect of feed flow rate on the content and recovery of α-terpineol. Bars with different letters are significantly different at *p* < 0.05.

Fig. 6 shows that feed flow rate strongly influenced both the α-terpineol content and recovery. At a flow rate of 1.0 mL min^−1^, efficient evaporation resulted in a high recovery (88.9%) but only moderate purity (67.7%) due to the co-evaporation of other volatile compounds. Increasing the flow rate to 1.5 mL min^−1^ maximized the α-terpineol content (70.8%) while maintaining a high recovery of 89.3%, indicating an optimal balance between purity and recovery. Beyond this point, higher flow rates (2.0–3.0 mL min^−1^) led to progressively lower contents (66.1–53.0%) and a pronounced decline in recovery, reaching 51.1% at 3.0 mL min^−1^. This decrease can be attributed to shorter residence times and thicker liquid films at elevated flow rates, which hinder uniform spreading and limit heat transfer on the evaporator surface.^36^ Unlike pressure and temperature, which caused only minor variations in recovery, feed flow rate directly affected film hydrodynamics and had the most significant impact on separation efficiency.

Comparable observations have been reported in previous studies on the molecular distillation of natural compounds. Ito *et al.* (2007) demonstrated that low feed flow rates combined with elevated evaporator temperatures enhanced free fatty acid removal and increased tocopherol concentration from soybean oil deodorizer distillate.^37^ Likewise, Fregolente *et al.* (2005) observed that excessively high feed flow rates led to reduced separation efficiency of monoglycerides due to inadequate heat transfer and incomplete evaporation.^38^ Taken together, these findings indicate that feed flow rates in the range of 1–2 mL min^−1^ represent an appropriate operating window for further optimization.

### Optimization of distillation conditions using response surface methodology

Although single-factor experiments provided a preliminary understanding of the effects of pressure, evaporator surface temperature, and feed flow rate on α-terpineol content and recovery, this approach does not account for potential interactions among these variables. In practice, operating parameters act simultaneously, and their combined effects can substantially influence separation efficiency. To address this limitation, response surface methodology (RSM) was applied as a statistical tool to model factor interactions and determine optimal operating conditions for enhancing α-terpineol enrichment and recovery.^39,40^

#### α-Terpineol content in the light fraction: experimental design and optimization using RSM-CCD

Based on the results of preliminary single-factor experiments, three operating parameters were identified as the key factors influencing the molecular distillation of the niaouli oil residue (NOR): absolute pressure (Pa) (*X*_1_), evaporator surface temperature (°C) (*X*_2_), and feed flow rate (mL min^−1^) (*X*_3_). Response surface methodology (RSM) combined with a central composite design (CCD) was applied to investigate the interactive effects of these variables and to determine the optimal conditions for α-terpineol enrichment in the light fraction. A total of 17 experimental runs were performed according to the CCD matrix, and the corresponding results are summarized in Table 1.

**Table 1:** α-Terpineol content in the light fraction based on the central composite design of MD

Ex	Coded variables			Pressure *X*_1_	Evaporator surface temperature *X*_2_	Feed flow rate *X*_3_	α-Terpineol content (%)
	*X*_1_ (Pa)	*X*_2_ (°C)	*X*_3_ (mL min^−1^)				
1	−1	−1	−1	150	30	1	65.92
2	1	−1	−1	250	30	1	66.22
3	−1	1	−1	150	40	1	68.35
4	1	1	−1	250	40	1	64.53
5	−1	−1	1	150	30	2	58.41
6	1	−1	1	250	30	2	68.43
7	−1	1	1	150	40	2	66.69
8	1	1	1	250	40	2	68.12
9	−1.68	0	0	116	35	1.5	59.46
10	1.68	0	0	284	35	1.5	66.17
11	0	−1.68	0	200	26.59	1.5	66.16
12	0	1.68	0	200	43.4	1.5	68,14
13	0	0	−1.68	200	35	0.66	69.88
14	0	0	1.68	200	35	2.34	66.66
15	0	0	0	200	35	1.5	70.87
16	0	0	0	200	35	1.5	70.61
17	0	0	0	200	35	1.5	70.55

The preliminary experiments defined the effective ranges of the process parameters, thereby narrowing the experimental domain and providing a basis for subsequent optimization using response surface methodology (RSM). Analysis of variance (ANOVA) was performed to evaluate the adequacy of the second-order polynomial model. The model was statistically significant (*p* < 0.05), with an Adeq Precision value of 15.126 (>4), an *R*^2^ of 0.9605, and an adjusted *R*^2^ of 0.9098, indicating a satisfactory fit and adequate predictive capability. After eliminating statistically non-significant terms at the 95% confidence level, the relationship between the response variable and the independent variables was expressed by the following second-order polynomial equations.

Regression eqn (5) in coded variables:

5 *Y* = 70.67 + 1.41*X*_1_ + 0.882*X*_2_ − 1.59*X*_1_*X*_2_ +1.87*X*_1_*X*_3_ + 0.904*X*_2_*X*_3_ − 2.77*X*_1_^2^ − 1.24*X*_2_^2^ − 0.843*X*_3_^2^

Regression eqn (6) in actual variables:

6 *Y* = −54.908 + 0.582*X*_1_ + 4.375*X*_2_ − 0.006*X*_1_*X*_2_ + 0.0745*X*_1_*X*_3_ + 0.362*X*_2_*X*_3_ − 0.001*X*_1_^2^ − 0.050*X*_2_^2^ − 3.373*X*_3_^2^

Within the investigated range, eqn (5) and (6) indicate that the response variable (*Y*) is primarily influenced by the linear terms of *X*_1_ (vacuum pressure) and *X*_2_ (evaporator surface temperature), the quadratic terms of all three variables (*X*_1_, *X*_2_, and *X*_3_), as well as the interaction terms *X*_1_*X*_2_, *X*_1_*X*_3_, and *X*_2_*X*_3_. These results demonstrate that α-terpineol enrichment depends on the combined effects of all three operating parameters, which is consistent with the trends observed in the single-factor experiments. Accordingly, the inclusion of vacuum pressure, evaporator surface temperature, and feed flow rate as independent variables in the optimization study is statistically justified.

The F-test and corresponding *p*-values (*p* < 0.05) indicated that the developed model was statistically significant and adequately described the experimental data. The coefficient of determination (*R*^2^ = 0.9605) showed that 96.05% of the variation in α-terpineol content could be explained by the combined effects of the independent variables, while only 3.95% was attributed to unexplained variability. Because *R*^2^ alone may overestimate model performance, the adjusted *R*^2^ (0.9098) was also considered, confirming the good agreement between the experimental and predicted values and demonstrating the adequacy of the model (Table 2).

**Table 2:** Model adequacy statistics for α-terpineol content

Response variable	Residue variance	*R* ^2^	Adjusted *R*^2^	C.V. (%)
α-Terpineol content	1.05	0.9605	0.9098	1.57

#### Optimization analysis through 3D response surface modeling

**Fig. 7:**
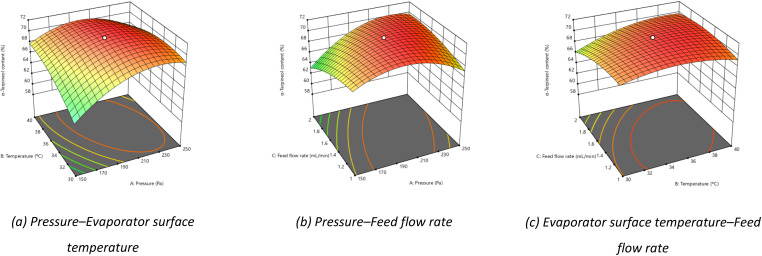
3D response surface models showing the effects of factor pairs on α-terpineol content.

Response surface models describe the variation of the response variable within the investigated experimental domain, enabling identification of optimal operating conditions. The interaction effects between factor pairs—pressure and evaporator surface temperature, pressure and feed flow rate, and evaporator surface temperature and feed flow rate—were found to significantly influence the α-terpineol content. As illustrated in Fig. 7, the α-terpineol content increased as the operating pressure rose from 150 to approximately 210 Pa, followed by a decline at higher pressures. In contrast, feed flow rates exceeding 2 mL min^−1^ resulted in a pronounced reduction in the response. These behaviors are consistent with the single-factor results and can be attributed to variations in molecular mean free path, compound interactions under high-vacuum conditions, and changes in liquid film uniformity on the evaporator surface.^33,41^

#### Determination of optimal operating conditions

**Fig. 8:**
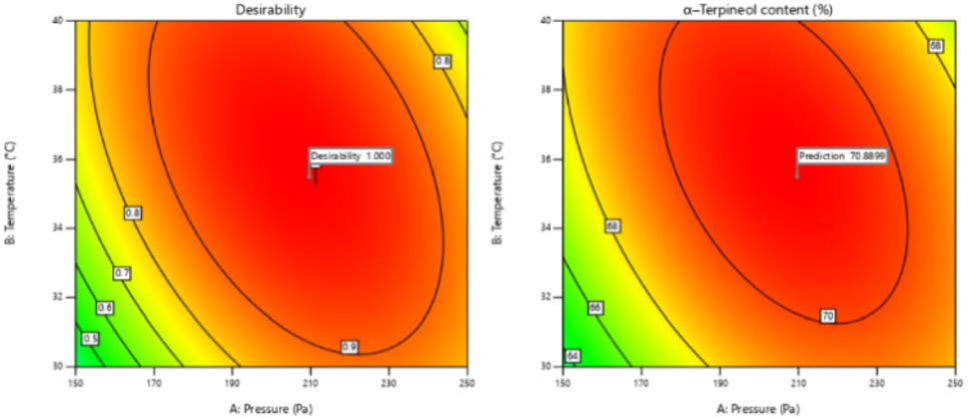
2D plot showing the effect of pressure and evaporator surface temperature on α-terpineol content at a feed flow rate of 1.39 mL min^−1^.

Based on the response surfaces shown in Fig. 8, the optimal operating conditions for enhancing α-terpineol content were identified as a pressure of 209.7 Pa, an evaporator surface temperature of 35.4 °C, and a feed flow rate of 1.39 mL min^−1^, with a predicted value of 70.89%. To validate the model prediction, experiments were conducted in triplicate under these conditions, and the results are summarized in Table 3. The maximum α-terpineol content obtained experimentally was 70.97%, accompanied by a recovery of 89.2%. The close agreement between the experimental and predicted values confirms the reliability and predictive capability of the RSM model.

**Table 3:** Validation of the optimum operating condition

	Experimental results			Mean value	Predicted value	Deviation (%)
α-Terpineol content	70.97	70.25	70.13	70.45	70.89	0.62

Overall, optimization using response surface methodology (RSM) identified a pressure of 209.7 Pa, an evaporator surface temperature of 35.4 °C, and a feed flow rate of 1.39 mL min^−1^ as the optimal operating conditions for the molecular distillation of Melaleuca quinquenervia essential oil residue. Under these conditions, the α-terpineol content in the light fraction reached 70.97%, with a recovery of 89.2%, demonstrating a marked improvement compared to the results obtained from preliminary single-factor experiments. Although the optimized process requires precise control of operating parameters, it enables more efficient separation and higher enrichment of α-terpineol, thereby highlighting the effectiveness of RSM as a tool for process optimization.

These results demonstrate that the integration of molecular distillation with statistical optimization provides an effective approach for enriching thermally sensitive oxygenated terpenoids from essential oil residues. Such compounds typically exhibit relatively high boiling points and are prone to thermal degradation under conventional distillation conditions. Molecular distillation, operating under high vacuum and short residence time, therefore offers favorable conditions for their selective enrichment.

More broadly, this approach may be applicable to the recovery and enrichment of other high-value natural compounds with similar physicochemical properties, particularly high-boiling bioactive constituents present in essential oil residues and related natural mixtures. In addition, the process offers environmental advantages because it avoids the use of organic solvents commonly required in liquid–liquid extraction, chromatographic separation, or crystallization processes. Furthermore, the relatively low operating temperature used in this study (35.4 °C) may help reduce energy consumption during large-scale operation.

### Changes in component profiles before and after MD

**Fig. 9:**
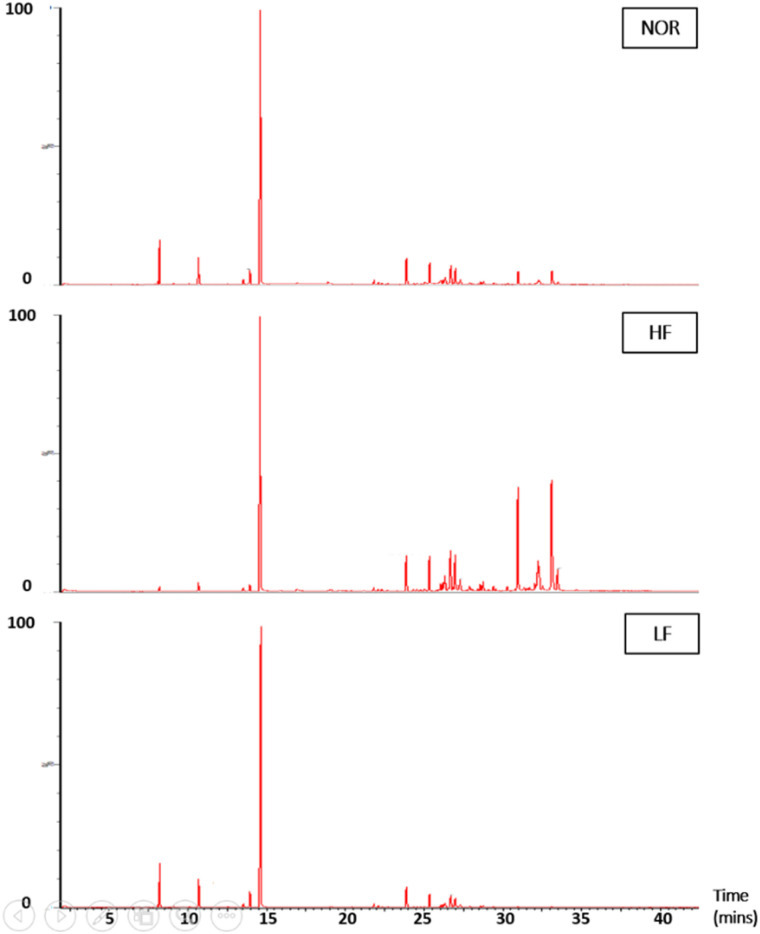
GC–MS chromatograms of the NOR, HF and LF obtained after MD.

Following RSM optimization, the chemical compositions of the NOR and the corresponding light and heavy fractions were compared to elucidate component distribution during molecular distillation. As shown in Table 4 and Fig. 9, the NOR—obtained after vacuum fractional distillation for eucalyptol recovery—contained only a relatively low level of eucalyptol (6.93%), indicating that most of this light component had been removed in the upstream process. Consistently, light monoterpenes were largely depleted, with only a trace amount of limonene detected (0.38%). In contrast, oxygenated monoterpenes with higher boiling points remained at appreciable levels, among which α-terpineol accounted for 36.52%, highlighting the NOR as a promising source for the recovery of biologically valuable compounds. In addition, the NOR contained considerable amounts of sesquiterpenes and oxygenated sesquiterpenes, including β-caryophyllene, α-humulene, β-selinene, guaiol and eudesmol.

**Table 4:** Chemical composition of the NOR and MD fractions

No.	Retention time (min)	Compounds	Retention index	Percentage (%)		
				NOR	LF	HF
**Monoterpene**						
1	8.085	d-Limonene	1031	0.38	0.41	0.28

**Oxygenated monoterpene**						
2	8.202	Eucalyptol	1032	6.93	6.65	—
3	10.091	Terpinolen	1088	0.29	0.23	—
4	10.676	Linalool	1099	6.17	5.13	0.84
5	13.518	δ-Terpineol	1166	0.94	1.01	0.28
6	13.936	Terpene-4-ol	1177	2.94	3.13	0.62
**7**	**14.588**	**α-Terpineol**	**1189**	**36.52**	**66.10**	**19.54**
8	16.945	Nerol	1222	0.83	0.51	0.27
9	19.001	Geraniol	1253	1.57	0.53	0.29

**Sequiterpene**						
10	21.81	Ylangene	1372	0.83	0.91	0.43
11	22.09	Copaene	1376	0.50	0.49	0.29
12	22.31	Geranyl acetate	1381	0.33	0.39	—
13	22.696	β-Elemene	1391	0.19	0.32	—
14	23.866	β-Caryophyllen	1419	6.81	3.86	4.52
15	24.317	γ-Elemene	1433	0.26	0.11	0.10
16	24.551	α-Guaiene	1439	0.31	0.16	0.12
17	25.036	β-Guaiene	1445	0.97	0.21	0.19
18	25.337	α-Humulene	1454	5.21	2.69	4.73
19	26.223	Patchoulene	1467	1.04	0.61	1.74
20	26.34	α-Amorphene	1483	1.53	1.87	2.26
21	26.691	β-Selinene	1486	5.56	1.05	7.31
22	26.992	α-Selinene	1494	3.29	1.02	5.33
23	27.293	δ-Cadinene	1524	1.58	0.11	3.07
24	27.895	Selina-3,7(11)-diene	1542	0.57	0.10	1.75
25	28.58	Guaia-3,9-diene	1556	0.55	0.10	2.02

**Oxygenated sequitepene**						
26	30.954	Guaiol	1596	3.04	—	12.59
27	32.224	γ-Eudesmol	1626	2.07	—	6.55
28	33.11	β-Eudesmol	1649	3.84	—	13.98
29	33.495	Bulnesol	1667	0.88	—	3.95
**Total identified components**				**95.93**	**97.70**	**93.05**

Molecular distillation resulted in a marked enrichment of α-terpineol in the light fraction, increasing from 36.52% in the niaouli oil residue to 66.10%. However, due to the close boiling points of oxygenated monoterpenes, most of these compounds were co-distilled into the LF, thereby limiting the selectivity of MD for their complete separation. Only trace amounts of sesquiterpenes and virtually no oxygenated sesquiterpenes were detected in this fraction, consistent with the expected volatility-based separation behavior. These trends are further supported by the GC–MS chromatogram (Fig. 9), in which α-terpineol appears as the dominant peak at approximately 14.6 min, accompanied by minor early-eluting monoterpenes and only a few low-intensity sesquiterpene peaks at longer retention times.

In contrast, the heavy fraction was dominated by high-boiling sesquiterpenes and oxygenated sesquiterpenes, particularly guaiol, eudesmol, and bulnesol—compounds associated with high biological value and characteristic woody aromas. This distribution is consistent with the GC–MS chromatogram (Fig. 9), which exhibits intense peaks in the 30–35 min retention time region, typical of oxygenated sesquiterpenes. The HF also retained 19.64% α-terpineol, likely due to the high viscosity of the residue, which hindered efficient evaporation and resulted in partial retention of this relatively volatile compound. Overall, these results demonstrate that molecular distillation effectively enriched α-terpineol in the light fraction while concentrating valuable oxygenated sesquiterpenes in the heavy fraction, confirming the potential of this technique for the selective recovery of functional compounds from NOR.

### Biological activity evaluation

#### Evaluation of antibacterial activity

The antibacterial activity of the α-terpineol-enriched light fraction was evaluated to examine its biological potential. Derived from the niaouli oil residue (NOR), this fraction is rich in α-terpineol, which has been widely reported to exhibit antimicrobial activity.

The agar disk diffusion assay (Table 5) revealed that all tested samples—including NOR, pure α-terpineol, LF, and ampicillin as a positive control—exhibited inhibitory activity against the five bacterial strains (*Staphylococcus aureus*, *Escherichia coli*, *Vibrio parahaemolyticus*, *Klebsiella pneumoniae*, and *Pseudomonas aeruginosa*). Among these, pure α-terpineol showed the strongest antibacterial activity, particularly against *S. aureus* (21.85 mm) and *K. pneumoniae* (20.16 mm). The α-terpineol-enriched light fraction also demonstrated notable antibacterial efficacy, especially against *P. aeruginosa*, a strain commonly associated with multidrug resistance. However, for certain strains (*S. aureus* and *E. coli*), the inhibitory effect of the LF was lower than that of pure α-terpineol, suggesting a close relationship between antibacterial activity and α-terpineol concentration. Importantly, the LF exhibited significantly higher antibacterial activity than the NOR and, in several cases, showed inhibition zones comparable to those of ampicillin, highlighting the practical value of molecular distillation for producing bioactive essential oil fraction.

**Table 5:** Diameter of inhibition zones of fractionated essential oil samples against pathogenic microorganisms

Diameter of inhibition zone (mm)				
	Ampicillin	NOR	α-Terpineol	LF
*S. aureus*	17.63 ± 0.42	15.41 ± 0.15 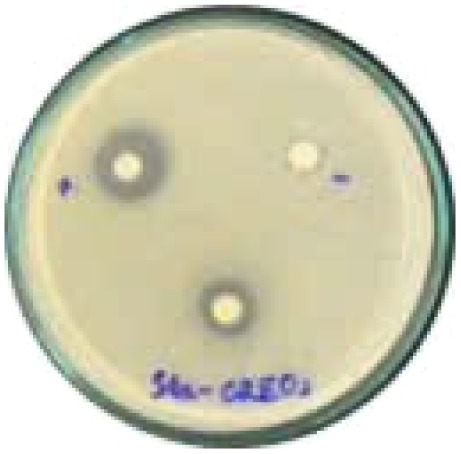	21.85 ± 0.05 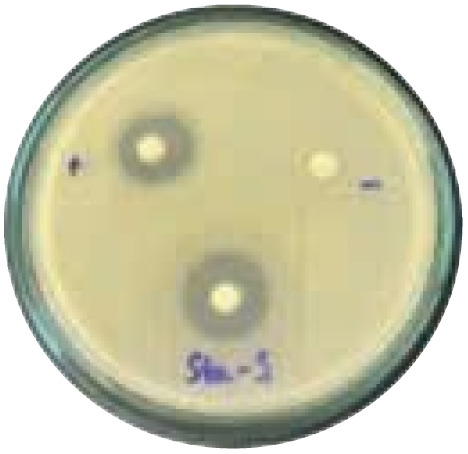	15.47 ± 0.07 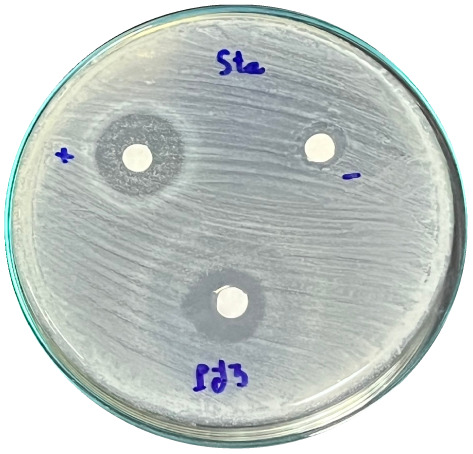
*E. coli*	15.75 ± 0.09	18.55 ± 0.46 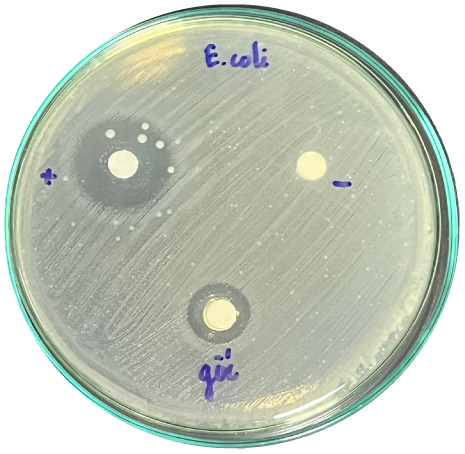	16.54 ± 0.25 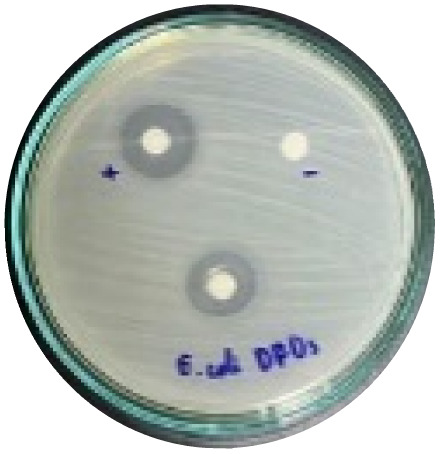	16.09 ± 0.50 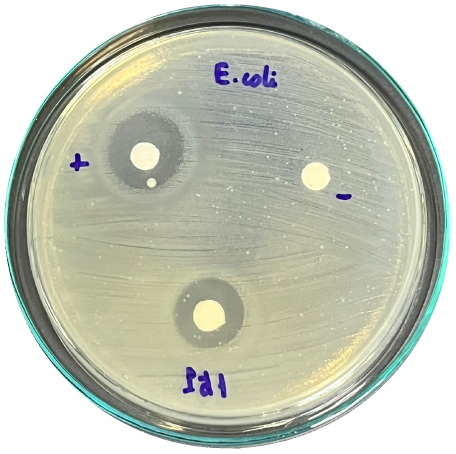
*V. parahaemolyticus*	15.41 ± 0.36	13.24 ± 0.20 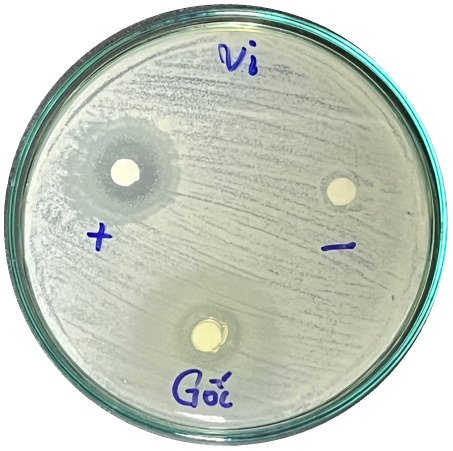	15.05 ± 0.39 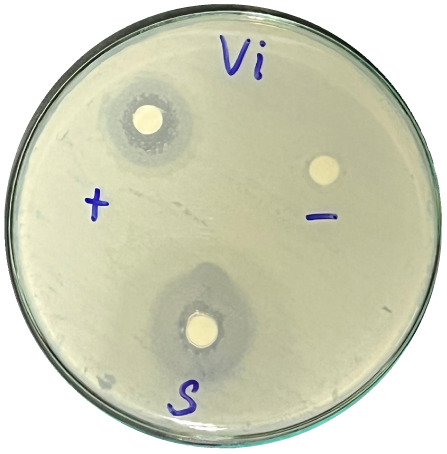	15.42 ± 0.18 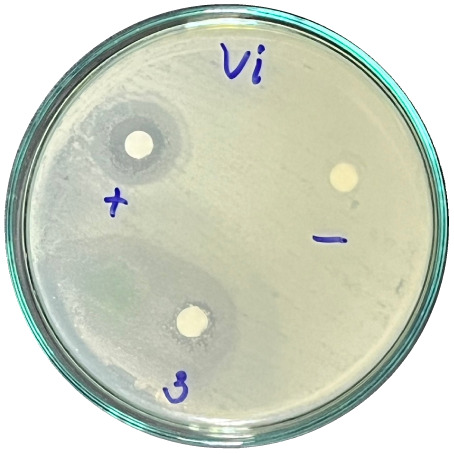
*K. pneumoniae*	25.18 ± 0.32	15.44 ± 0.48 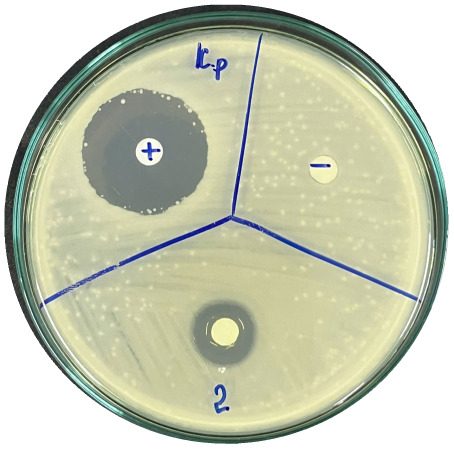	20.16 ± 0.04 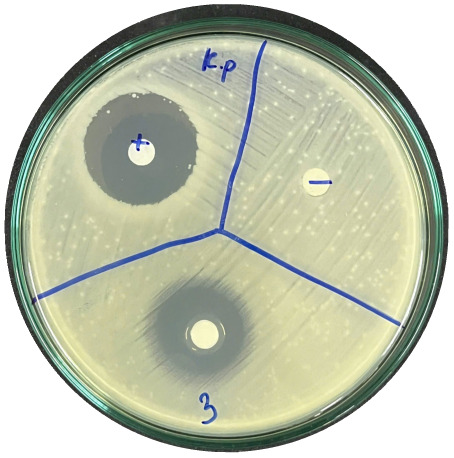	19.68 ± 0.18 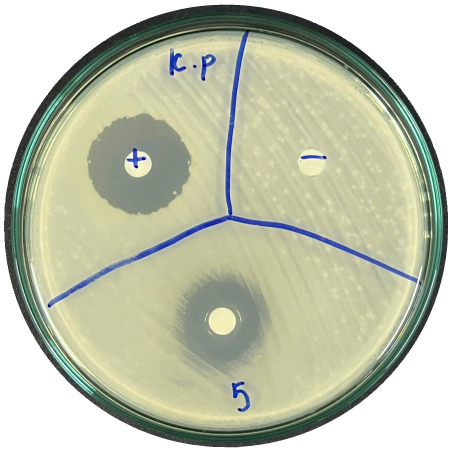
*P. aeruginosa*	20.98 ± 0.61	8.46 ± 0.37 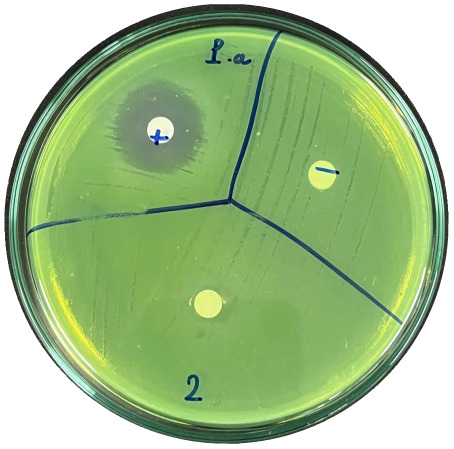	10.72 ± 0.15 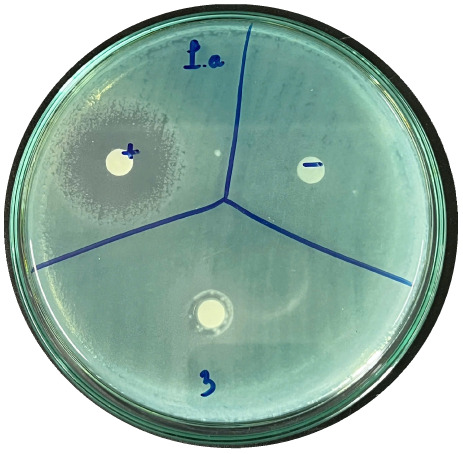	11.79 ± 0.10 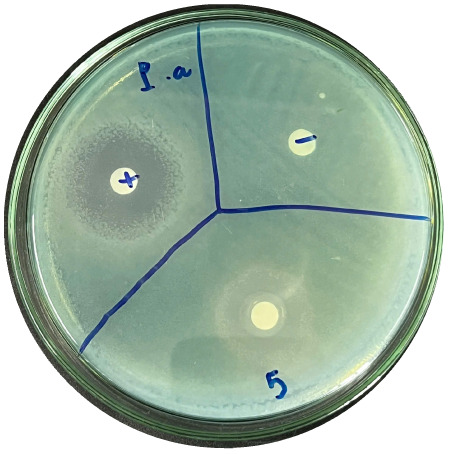

#### Evaluation of anti-inflammatory activity

The NO inhibitory effects of all samples increased in a concentration-dependent manner. The anti-inflammatory activity, expressed in terms of IC_50_ values, differed markedly among the samples (Table 6). Among all samples, pure α-terpineol exhibited the strongest anti-inflammatory activity, with an IC_50_ of 41.84 ± 1.39 µg mL^−1^, confirming its role as the principal active component. In contrast, the niaouli oil residue (NOR) showed the weakest activity, with an IC_50_ of 329.16 ± 7.29 µg mL^−1^.

**Table 6:** NO inhibition (%) and IC_50_ (µg mL^−1^) of test samples

Sample conc^a^	NO inhibition (%) at different concentration (µg mL^−1^)								IC_50_ value
	50	100	150	200	400	600	800	1000	
NOR	16.26 ± 0.61	26.17 ± 0.87	32.72 ± 0.81	41.53 ± 0.63	51.2 ± 0.07	58.52 ± 0.12	67.39 ± 0.64	76.01 ± 0.39	329.16 ± 7.29
LF	37.59 ± 0.45	41.4 ± 0.69	51.45 ± 1.46	58.64 ± 1.24	67.27 ± 0.17	69.08 ± 0.43	73.03 ± 0.29	81.33 ± 0.09	135.01 ± 4.51
HF	31.02 ± 1.07	37.07 ± 0.22	44.39 ± 0.16	57.49 ± 0.13	64.09 ± 0.46	80.1 ± 0.38	81.98 ± 0.21	85.23 ± 0.72	161.23 ± 3.10
α-Terpineol	52.49 ± 0.35	59.75 ± 0.73	65.97 ± 0.35	73.75 ± 0.04	81.79 ± 0.39	84.38 ± 0.31	85.68 ± 0.13	86.71 ± 0.1	41.84 ± 1.39

a Conc., concentration (µg mL^−1^).

Molecular distillation significantly enhanced the anti-inflammatory effect of the residue, as reflected by the heavy fraction (HF; IC_50_ = 161.23 ± 3.10 µg mL^−1^) and, more notably, the α-terpineol-enriched light fraction (LF), which exhibited a lower IC_50_ value of 135.01 ± 4.51 µg mL^−1^. The superior activity of the LF compared to the NOR and the HF is consistent with its elevated α-terpineol content. Overall, these results demonstrate that molecular distillation effectively enhances the anti-inflammatory potential of niaouli oil residue by concentrating α-terpineol into a bioactive fraction.

The observed anti-inflammatory activity of the α-terpineol-enriched fractions may be interpreted based on previously reported mechanistic studies on α-terpineol. Molecular docking studies have suggested that α-terpineol can interact directly with inducible nitric oxide synthase (iNOS) through hydrogen bonding and steric interactions within the enzyme active site. In particular, the hydroxyl group of α-terpineol has been reported to form a hydrogen bond with the THR324 residue of iNOS, which may contribute to the inhibition of nitric oxide production. Such interactions may partly account for the stronger NO inhibitory activity observed for the α-terpineol-enriched LF fraction.^42^

The light fraction not only exhibited superior activity compared to the other fractions but also contained a high proportion of α-terpineol, approaching the purity of the isolated compound. In addition to its anti-inflammatory efficacy, the LF also exhibited high biocompatibility, showing the highest cell viability (from 68.58 ± 0.27 to 103.87 ± 0.97) among all fractions and even a tendency to promote cell proliferation at low concentrations 25–100 (µg mL^−1^). Notably, at the IC_50_ level, the cell viability of all samples remained above 70% (Table 7), indicating that the observed anti-inflammatory effects were achieved without significant cytotoxicity. These results confirm that enrichment of α-terpineol by molecular distillation substantially enhances the anti-inflammatory potential of the refined fractions.

**Table 7:** Cell viability (%) of the samples after anti-inflammatory treatment

Sample conc^a^	Cell viability (%) at different concentrations (µg mL^−1^)								
	25	50	100	150	200	400	600	800	1000
α-Terpineol	100 ± 1	94.6 ± 0.48	89.63 ± 0.42	86.17 ± 0.72	79.44 ± 0.25	78.17 ± 0.16	68.25 ± 0.14	61.74 ± 0.49	55.24 ± 0.36
NOR	—	96.47 ± 0.37	90.8 ± 0.44	86.05 ± 0.47	79.77 ± 0.3	72.88 ± 0.73	67.92 ± 0.26	60.81 ± 0.19	56.78 ± 0.51
HF	—	89.2 ± 0.53	84.23 ± 0.08	77.68 ± 0.76	75.31 ± 0.81	67.48 ± 0.59	63.01 ± 0.4	59.37 ± 0.32	54.79 ± 0.32
LF	—	103.87 ± 0.97	97.14 ± 0.96	92.01 ± 0.85	87.22 ± 1.31	81.32 ± 1.22	78.12 ± 0.61	73.16 ± 0.51	68.58 ± 0.27

a Conc., concentration (µg mL^−1^).

## Conclusion

This study demonstrates the potential of *Melaleuca quinquenervia* essential oil residue as a valuable source of α-terpineol, showing that molecular distillation can effectively convert an underutilized by-product into a bioactive fraction. Through the integration of response surface methodology, a systematic optimization strategy was established to maximize both α-terpineol enrichment and recovery. The results confirm the effectiveness of molecular distillation not only as a separation method but also as a practical means for upgrading essential oil residues into value-added resources. Moreover, the proposed strategy provides an applicable approach for the sustainable valorization of high-boiling compounds from various essential oils.

## Author contributions

Dinh-Nhat Do, Minh-Tinh Trinh-Le and Hoang-Yen Nguyen-Thi carried out the experiments and data analysis. Minh-Chau Pham-Vu revised, refined the manuscript and coordinated the submission process. Tan-Viet Tran and Xuan-Tien Le provided conceptual guidance and supervision, with final review and approval by Xuan-Tien Le.

## Conflicts of interest

There are no conflicts to declare.

## Data Availability

The data supporting the findings of this study are available within the article.
